# S1 domain of the porcine epidemic diarrhea virus spike protein as a vaccine antigen

**DOI:** 10.1186/s12985-016-0512-8

**Published:** 2016-04-01

**Authors:** Niraj Makadiya, Robert Brownlie, Jan van den Hurk, Nathalie Berube, Brenda Allan, Volker Gerdts, Alexander Zakhartchouk

**Affiliations:** Vaccine and Infectious Disease Organization - International Vaccine Center (VIDO-InterVac), University of Saskatchewan, 120 Veterinary Road, Saskatoon, SK S7N 5E3 Canada

**Keywords:** PEDV, S1, Subunit vaccine, Lactogenic immunity

## Abstract

**Background:**

Porcine epidemic diarrhea virus (PEDV) is a highly contagious virus infecting pigs of all ages with high morbidity and mortality among newborn piglets. Currently, there is no effective vaccine available to protect the pigs from PEDV. The N-terminal subunit of spike protein (S1) is responsible for virus binding to the cellular receptor and contains a number of neutralizing antibody epitopes. Thus, we expressed and produced recombinant S1 protein to protect newborn piglets by immunization of sows.

**Methods:**

Affinity tagged PEDV S1 protein was expressed in a secretory form in yeast, insect and mammalian cells to identify the most suitable production system. Purified recombinant protein was analysed by SDS-PAGE, Western blot and deglycosylation assay. A pregnant sow was intramuscularly immunized three times with adjuvanted recombinant protein prior to farrowing. PEDV-specific immune responses in sera and colostrum of the sow and piglets were assayed by ELISA and virus neutralization assays. Piglets were challenged orally with PEDV, and clinical parameters were monitored for 6 days post-challenge.

**Results and conclusion:**

Of three eukaryotic expression systems tested (yeast, insect-cell, and mammalian), expression by HEK-293 T cells gave the highest yield of protein that was N-glycosylated and was the most appropriate candidate for vaccination. Administration of the subunit vaccine in a sow resulted in induction of S1-specific IgG and IgA that were passively transferred to the suckling piglets. Also, high virus neutralization titres were observed in the serum of the vaccinated sow and its piglets. After PEDV challenge, piglets born to the vaccinated sow exhibited less severe signs of disease and significantly lower mortality compared to the piglets of a control sow. However, there were no significant differences in diarrhea, body weight and virus shedding. Thus, vaccination with S1 subunit vaccine failed to provide complete protection to suckling piglets after challenge exposure, and further improvements are needed for the development of a subunit vaccine that fully protects against PEDV infection.

## Background

Porcine epidemic diarrhea virus (PEDV) is an enveloped, positive-stranded RNA virus which readily infects pigs, resulting in highly contagious porcine epidemic diarrhea [1]. PEDV belongs to family *Coronaviridae*, subfamily *Coronavirinae* and genus *Alphacoronavirus* [2]. Some viruses of the *Coronaviridae* family cause severe disease in humans such as severe acute respiratory syndrome coronavirus (SARS-CoV) and Middle East respiratory syndrome coronavirus (MERS-CoV) [3, 4]. Coronaviruses of veterinary significance include avian infectious bronchitis virus infecting chickens, transmissible gastroenteritis virus (TGEV) infecting pigs, bovine coronavirus, feline coronaviruses, canine coronavirus and turkey coronavirus [5].

Porcine epidemic diarrhea (PED) was first observed in Europe in the early 1970s, and PEDV was first isolated in Belgium in 1978 [6]. Subsequently, PED has become an endemic disease in Asian pig farming countries. Severe PED outbreaks were reported in China in 2010–2012 [7, 8]. From April 2013 to the present, major PEDV outbreaks have been reported in the USA [9], Canada [10], Taiwan [11] and Europian countries [12, 13]. The PED is characterized by the presence of watery diarrhea in the infected piglets in first few weeks of their life, dehydration, vomiting and anorexia resulting in high morbidity and mortality [14]. PEDV infection of older pigs results in considerably lower morbidity and mortality. The symptoms of the disease are similar to transmissible gastroenteritis of pigs and hence only laboratory tests can aid in differencial diagnosis [15]. Although, some efforts have been made to create the vaccine against PEDV with varied success, no effective vaccine is available in the market to protect the newborn piglets [14, 15].

The size of PEDV genomic RNA is about 28 kb, and contains seven open reading frames (ORFs) encoding viral proteins: 1A, 1B, spike (S), ORF3, envelope (E), membrane (M) and nucleocapsid (N). The S protein is present at the outer surface of the virion and is 1386 amino acid long [16]. The spike protein of coronaviruses forms trimers and plays an important role in the virus attachment and in virus-cell membrane fusion [17]. Porcine aminopeptidase N has been demonstrated to be a functional receptor for the PEDV coronavirus [18]. The S protein of PEDV is a class I membrane glycoprotein consisting of two subunits: the N-terminal S1 and the C-terminal S2. Cleavage of spike protein into S1 and S2 is an essential event in the cellular entry for wild-type PEDV virus but not for cell culture adapted PEDV [19]. Proteolytic cleavage of spike protein in PEDV needs trypsin [19, 20]. Several neutralizing epitopes have been identified on the S protein sequence [21–23], and the recombinant S1 protein was previously shown to have protective activity in piglets [24].

## Results and discussion

### Expression of S1 in yeast cells

**Fig. 1:**
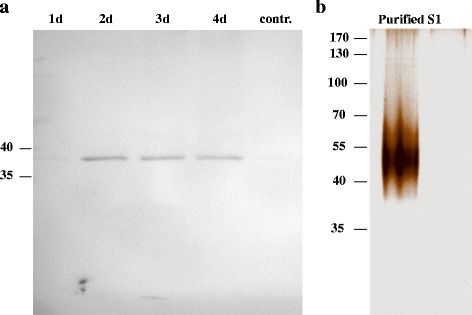
Expression of the recombinant PEDV S1 protein in PichiaPink *Pichia pastoris*. **a** A small scale S1 protein expression experiment was performed, and samples of culture medium were collected on indicated days post-induction and detected by Western blotting using anti-his antibody. **b** A large scale S1 protein purification was performed from the yeast cell supernatant at day 4 post-induction, and the purified protein was anyalzed by staining of SDS-PAGE by silver nitrate. Medium from the un-induced cells served as a negative control. Numbers on the left indicate the protein molecular marker size in kDa

Initial attempts to express the S1 protein in the bacterial cells were not successful (data not shown), which may be due to problems in processing of the S1 protein in prokaryotic cells. Therefore, we used PichiaPink (*Pichia pastoris*) yeast cells to express S1 from a synthetic S1 gene codon optimized for yeast and containing a C-terminal histidine-tag to aid purification. Initially, the time course was performed for the expression of the S1 protein in the yeast cells over the period of 4 days. Western blot analysis of the cell culture medium of transformed yeast cells resulted in the detection of a specific 35–40 kDa band when probed with anti-his antibody (Fig. 1a). The observed protein molecular weight was less than the expected 80.9 kDa molecular weight of un-glycosylated S1. This may be due to the cleavage of the protein by yeast protease. The purified protein was detected in SDS-PAGE as a smearing band in a range of 40–70 kDa (Fig. 1b). The band pattern may be the result of glycosylation of multiple sites in the S1 protein by the yeast cells. The yield of the purified protein from one liter of yeast culture was found to be 180 μg.

### Expression of S1 by recombinant baculovirus

**Fig. 2:**
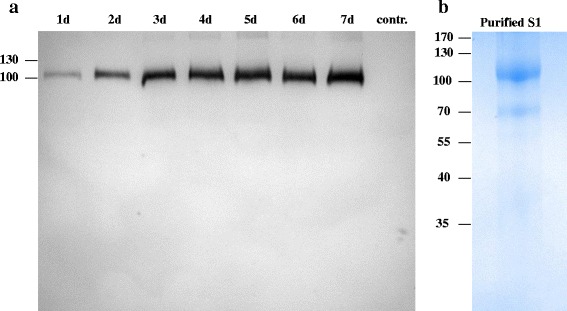
Expression of the recombinant PEDV S1 protein in insect Sf9 cells. **a** A small scale S1 protein expression experiment was performed by infecting Sf9 cells with recombinant baculovirus expressing PEDV S1 gene. Cell culture media were collected on indicated days post-infection and analysed by Western blotting using anti-his antibody. **b** Large scale S1 protein purification was performed on cell culture medium of the cells infected with recombinant baculovirus at day 7 post-infection, and the purified protein was anyalzed by SDS-PAGE and stained by Coomassie Blue. Medium from un-infected cells served as a negative control. Numbers on the left indicate the protein molecular marker size in kDa

To increase the PEDV S1 protein yield and to achieve a full-length S1 protein expression, a baculovirus expression system was employed. To this end, histidine-tagged PEDV S1 gene was cloned into a BACMID containing the genome of baculovirus, transfected into Sf9 cells and the recombinant baculovirus was recovered. Small scale PEDV S1 protein expression in the recombinant baculovirus infected cells was analysed by Western blotting using the anti-his antibody at days 1 to 7 post-infection (Fig. 2a). The S1 protein was detected as a distinct 100 kDa band and band intensity increased on each subsequent day. Size and integrity of affinity purified S1 from the cell culture medium of baculovirus infected cells was found to be of the same size (Fig. 2b) as detected earlier (Fig. 2a) with the yield of 3.9 mg/L.

### Expression of S1 in mammalian cells

**Fig. 3:**
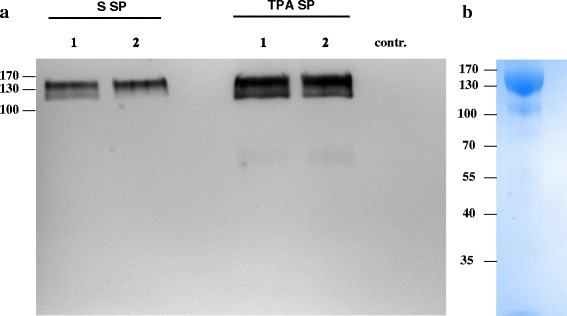
Expression of the recombinant PEDV S1 protein in HEK 293 T cells. **a** Cells were grown to confluency in 6 well plates. The DMEM was replaced with serum free medium (either SFM4HEK293; lane 1 or Opti-MEM; lane 2) and incubated further for 24 h before analyzing culture supernatant by Western blotting with anti-his antibody. The S1 was expressed with native signal peptide (S SP) or with TPA signal peptide (TPA SP). A negative control was HEK-293 T cells that had not been transfected. **b** Analysis of the purified S1 protein by Coomassie Blue staining of SDS-PAGE. Numbers on the left indicate the protein molecular marker size in kDa

Mammalian expression of S1 utilizing a human CMV promoter within an episomal vector was also investigated. In preliminary experiments, expression of S1 in which the native signal peptide was replaced with that of tissue plaminogen activator (TPA) was compared with expression of S1 containing native signal peptide. Transformed HEK-293 T cells were grown for 24 h in serum free medium, and the supernatant was then analysed by Western blotting for expression of histidine-tagged protein. The S1 protein was detected as a single 130 kDa band for both cells expressing S1 with native signal peptide and cells expressing S1 with TPA (Fig. 3a). However, the presence of TPA substantially enhanced expression of S1. Therefore, S1 with TPA was subsequently used for production and purification of the vaccine antigen. Affinity purified S1 protein was analysed on SDS-PAGE by Coomassie blue staining and found to be of the expected size (Fig. 3b). The yield of the S1 protein from HEK 293 T cell culture medium was found to be 30 mg/L, which was 10 and 100-fold higher than the yields obtained from insect cells or yeast cells respectively.

### Glycosylation profile of the purified S1 protein

As there are multiple glycosylation sites present in the spike proteins of other coronaviruses, the purified PEDV S1 protein was analysed for glycosylation. Sensitivity to glycosidases PNGase F (removes N-linked glycans) and *O*-glycosidase (removes O-linked glycans) was used to determine the nature of glycosylation of the recombinant protein.

**Fig. 4:**
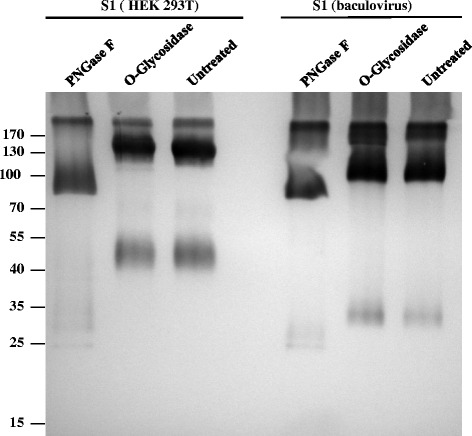
Deglycosylation analysis of the purified recombinant S1 proteins. S1 proteins purified from cell culture media of HEK 293 T cells or baculovirus infected Sf9 cells were treated with deglycosylation enzymes PNGase F or *O*-Glycosidase or left un-treated. Proteins were resolved on 10 % SDS-PAGE and analysed by Western blotting using anti-his antibody. Numbers on the left indicate the protein molecular marker size in kDa

PNGase F, but not *O*-glycosidase, increased the electrophoretic mobility of purified recombinant S1 produced by both HEK-293 T or baculovirus-infected cells (Fig. 4) suggesting that the recombinant protein is N- but not O-glycosylated when produced by either mammalian or insect cells.

### Humoral immune responses in sows

**Fig. 5:**
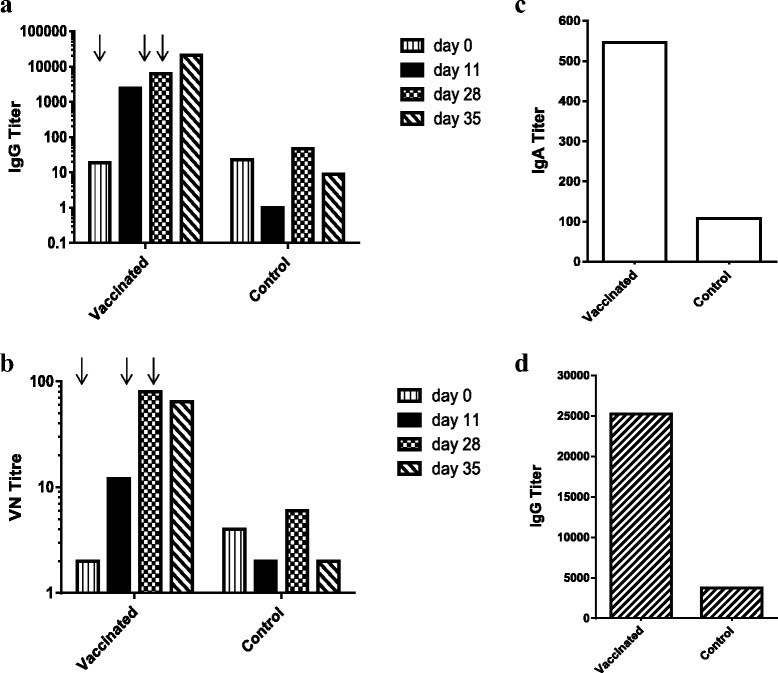
PEDV S1-specific antibody responses in sows. **a** Sow serum IgG titers were measured at the indicated time points post-vaccination by ELISA using the recombinant purified S1 protein as an antigen. Sows were vaccinated three times on days 0, 14 and 28 as indicated by arrows. **b** Virus-neutralizing antibody titers in sera from sows collected at indicated time points post-vaccination. **c** Colostrum IgA titers and **d** colostrum IgG titers were determinded by ELISA. Colostrum samples were collected on the day of farrowing

To determine the immunogenicity of the S1-based subunit vaccine, a pregnant sow was immunized intramuscularly with adjuvanted S1 recombinat protein produced from HEK 293 T cells. The humoral immune responses elicited by the subunit vaccine were examined by ELISA and serum neutralization assay. The vaccinated sow showed an increase in S1-specific serum IgG titer after the first, second and third vaccination (Fig. 5a) whereas no increase was noticed in the control sow. Furthermore, in contrast to the control sow, vaccinated sow demonstrated detectable serum neutralizing-antibody titers at day 11 post-vaccination, and by 28 days the titers had increased 100-fold (Fig. 5b). In contrast to ELISA titers (Fig. 5a), virus neutralizing titers did not increase after the third immunization of sow by day 35 (Fig. 5b).

Piglets that regularly suckle the immune mother receive colostrum/milk antibody, a process that cofers passive immunity to the piglets. Therefore, we tested colostrum samples collected from the vaccinated and control sows on the day of farrowing for the presense of PEDV S1-specific IgA and IgG antibody titres. The IgA and IgG titers were higher in colostrum of the vaccinated sow compared to the control sow (Fig. 5c and d). The IgG titer levels were 5-fold higher than IgA titer levels, but it is not surprising since IgG is the major isotype in sow colostrum whereas IgA predominates in milk [25].

### Humoral immune responses in piglets

**Fig. 6:**
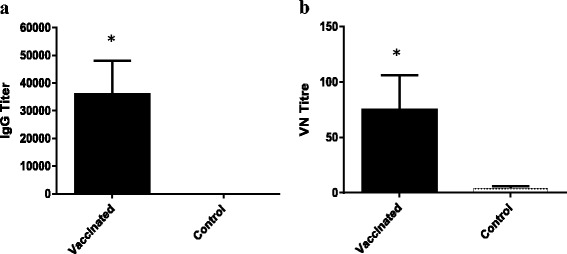
PEDV S1-specific antibody titers in piglets. **a** Piglet serum IgG titers were measured on the day of PEDV challenge by ELISA. The values represent the mean IgG titers of litters from a vaccinated sow (*n* = 8) and litters (*n* = 7) of an unvaccinated control sow. Error bars represent the standard deviation. **b** Virus-neutralizing antibody titers in sera from piglets at the day of PEDV challenge. The error bars represent the standard deviation of the mean. * means significantly different results (*P* < 0.001)

Sera of piglets, collected on day 4 after birth, had high titres of S1-specific IgG in the litter of a vaccinated sow, while no titres were found in the litter of a control sow (Fig. 6a). These data indicate that passive transfer of S1-specific IgG antibodies occurred between the vaccinated pregnant sow and the offspring via colostrum/milk. In addition, these specific antibodies also conferred virus neutralization (Fig. 6b).

### Virus shedding in challenged piglets

**Fig. 7:**
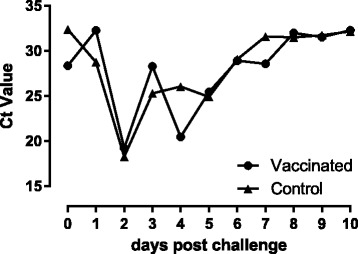
Virus shedding in piglets. PEDV viral RNA was extracted from rectal swabs of challenged piglets and assessed by real-time reverse transcription PCR

Virus shedding in feces of piglets collected at different time points post challenge was assessed by a PEDV N gene-based real-time RT-PCR, and the cycle threshold (*C*_*T*_) values are shown in Fig. 7. No differences were observed in PEDV shedding between groups of piglets of vaccinated and control sow.

### Clinical observations

**Fig. 8:**
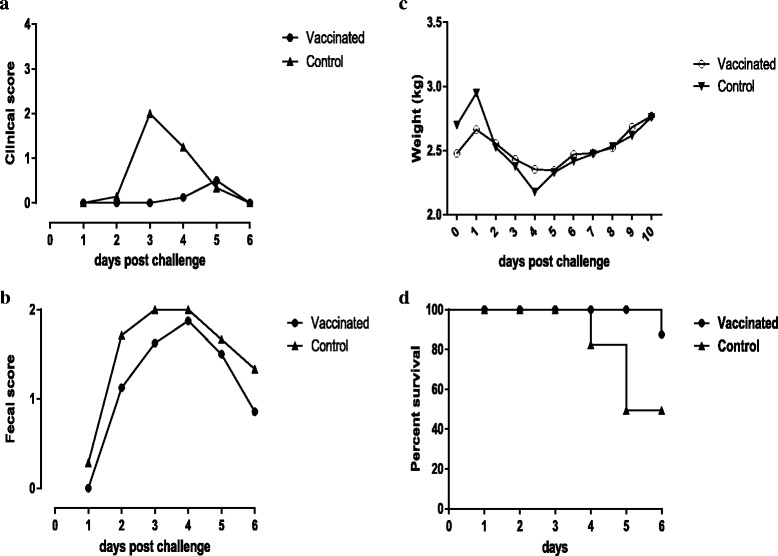
Protective efficacy of the vaccine. Litters from a vaccinated (*n* = 8) and a control sow (*n* = 7) were orally challenged with virulent PEDV at 4 days of age. Clinical (**a**), fecal scores (**b**), average weight (**c**) and survival of piglets (**d**) were evaluated

There were no adverse reactions noticed at the injection site or overall health of sow post-vaccination. Each day post-challenge, the piglets were evaluated by animal services veterinarian for the clinical scores. The clinical score was assigned as 0 (healthy) to 4 (dead or moribund) for each piglet based on parameters such as animal demeanor, degree of depression and willingness to nurse. The average clinical scores for the vaccinated sow’s piglets was close to zero, whereas the control sow’s piglets had average scores reaching on day 2 and 3 post-challenge (Fig. 8a). Similarly, fecal scores were recorded from 0 (normal pasty faces) to 2 (watery diarrhea) for all the piglets each day post-challenge. Diarrhea increased until day 4 post-challenge and then started to reduce for both groups of piglets (Fig. 8b).

The weight of each piglet was monitored every day post-farrowing for 10 days, and the analysis of the average weight of piglets showed no appreciable differences between two groups of piglets (Fig. 8c).

Survival of the piglets was monitored for 10 days after challenge. At 6 days post-challenge, 7 out of 8 piglets (87.5 %) of vaccinated sow survived whereas only 3 out of 7 piglets (42.9 %) of control sow survived the challenge (Fig. 8d). Statistically significant (*P* = 0.0002) better survival rate in the litter of vaccinated sow can be explained by the fact that these piglets were less depressed and showed more willingness to nurse than piglets of a control sow. However, more experiments with large number of sows are needed to confirm these data.

In summary, our vaccine had negligible effect in either preventing diarrhea or preventing PEDV-mediated weight loss, but did partially protect piglets in terms of severity of clinical disease and significantly reduced mortality. Future work to improve the protective efficacy of the subunit vaccine for PEDV may include testing new adjuvants. For instance, in the recently published report, oil-in-water adjuvant was used to formulate recombinant S1 protein [24]. Another approach is use of oral immunization instead of intramuscular (IM) route. A field study demonstrated that orally vaccinated sows with live attenuated PEDV vaccine exhibited higher IgA and virus neutralizing antibody levels in the colostrum or sera compared to those of the counterparts administered the IM vaccine with the same dose [26]. To deliver a recombinant protein orally, a live vector such as adenoviral vector might be used [27].

## Conclusions

Amongst three eukaryotic expression systems, the highest production of S1 was achieved using a mammalian expression with the TPA signal sequence. This method of expression can be used to produce large amount of S1 for vaccine, diagnostic and research purposes.

A sow vaccinated three times with the S1 subunit vaccine had high IgG titres and high virus neutralization titres in the serum as compared to the control sow. Also, high levels of IgG and IgA titers were found in the colostrum of the vaccinated sow. Furthermore, maternal transfer of antibody was demonstrated as only the serum of suckling piglets had high titres of S1 specific IgG and viral-neutralizing antibody. There was higher survival rate among of piglets of vaccinated sow (87.5 %) than in the piglets of control sow (42.9 %) after oral PEDV challenge (*P* = 0.0002). These survival rates suggest the potential of the S1 protein subunit vaccine in preventing piglet mortality. The vaccinated sow’s piglets exhibited lower clinical signs of disease except diarrhea throughout the six day post-challenge period than control piglets. Surprisingly, there was no difference in average weight and PEDV shedding among two groups of piglets. These observations suggest the inability of the S1 subunit vaccine in our experiment to control all PED clinical signs although more experiments involving a large number of sows are needed to confirm these findings.

## Methods

### Cells and virus

Human embryo kidney (HEK) 293 T (ATCC CRL-1573) and Vero 76 (ATCC CRL-1587) cells were cultured in Dulbecco’s modified Eagle medium (DMEM) supplemented with 50 μg/mL gentamycin and 10 % fetal bovine serum. PichiaPink Strain 1 (Invitrogen) was grown in YPD medium. Insect Sf9 cells (Gibco) were grown in Sf-900 serum-free medium (ThermoFisher Scientific).

PEDV strain USA/Colorado/2013 (GeneBank accession no. KF272920) was provided by Diagnostic Virology Laboratory (NVLS, Ames, USA), and the virus was propagated on Vero 76 cells in DMEM containing 50 μg/mL gentamycin, 2 μg/mL TPCK-trypsin and 0.2 % bovine serum albumin.

### Expression of recombinant S1 protein in yeast cells

Yeast codon optimized PEDV S1 gene (coding animo acids 1–734) with the 3’-end polyhistidine coding sequence was synthesized by GenScript. Synthetic S1 gene was digested with restriction enzymes *Mly*I-*Kpn*I and cloned into the pPinkα-HC vector (ThermoFisher Scientific) into *Stu*I-*Kpn*I sites. Expression of the recombinant protein was performed as recommended by manufacturers (ThermoFisher Scientific). Briefly, PichiaPink strain 1 (*ade2*^*−*^) was electroporated with pPinkα-S1 linearized plasmid DNA and plated on PAD (Pichia Adenine Dropout) agar plates for selecting transformants. After incubation at 30 °C for 5 days, white colonies were screened for expression of S1. Recombinant yeast cells were grown in buffered glycerol-complex medium (BMGY) and induction was performed in buffered methanol-complex medium (BMMY) with 0.5 % methanol. To prevent non-specific cleavage of S1 protein by yeast cell proteases, aprotinin and phenylmethylsulfonyl fluoride (PMSF) were added during the induction phase at 1 μg/mL each. Yeast cells were grown at 30 °C for 4 days and centrifuged at 3000 g for 5 min at room temperature and supernatant was collected for protein purification.

### Expression of recombinant S1 protein in insect cells

S1 coding region of PEDV genome was PCR amplified using primers (5’-TCCGATGAATTCGCCACCATGAAGTCACTCACCTATTTTTGG-3’ and 5’- CTAGATCTCGAGTCAGTGGTGATGATGGTGGTGGAAGCCAGGGAGTTCGCGG-3’). The resulting PCR product was cloned into the *Xho*I, *Eco*RI sites of pFastBac vector (ThermoFisher Scientific). Cellfectin® II Reagent (ThermoFisher Scientific) was used for transfecting Sf9 cells in Grace’s insect cell culture medium (ThermoFisher Scientific) for generating recombinant baculovirus. All the steps of generating the recombinant baculovirus full length genome, screening, rescue of recombinant baculovirus and large scale production of S1 protein in Sf9 cells was performed according to manufacturer’s recommendations for Bac-to-Bac Baculovirus Expression System (ThermoFisher Scientific). Briefly, the Sf9 cells were grown at 30 °C for 24 h in the Sf-900 II SFM (ThermoFisher Scientific) at an orbital shaker incubator in two 1 L flasks (500 mL culture volume). Sf9 cells were infected with the recombinant baculovirus at an MOI of 1 and kept for 7 days in the incubator for secretion of the recombinant S1 protein. The supernatant was collected by centrifuging the insect cells at 500 g for 5 min at room temperature.

### Expression of recombinant S1 protein in mammalian cells

The S1 ORF plus C-terminal his_10_ tag (codon optimized for mammalian expression) together with a proceeding Kozak sequence, was cloned downstream of a human CMV promoter plus intron, contained within an in-house episomal vector; elements downstream of the S1 ORF included a woodchuck hepatitis post-transcriptional regulatory element and bovine growth hormone poly-adenylation site (DNA sequence of constructs are available upon request). Constructs were transfected into HEK293T cells using Turbofect (ThermoFisher Scientific) according to the manufacturer’s instructions. Cells stably maintaining the episomal constructs were selected by puromycin. For protein production, stably transfected cells were grown in SFM4HEK293 medium (ThermoFisher Scientific) with shaking at 37^0^C and 5 % CO_2_. Supernatant was harvested and processed for purification.

### Purification of recombinant S1 protein

Purifications of supernatants from the recombinant yeast cells and recombinant baculovirus infected cells were performed in the same way. First, equal volume of wash buffer was added to the samples (50 mM sodium phosphate, 0.3 M sodium chloride, 10 mM imidazole pH 8.0) and then pH was adjusted to 8.0. Next, the samples were passed through 0.2 μm filters to remove cellular debris and applyed to the HisSelect Ni Affinity (Sigma Aldrich) column. The column was washed with three sample volumes of wash buffer, followed by protein elution in one sample volume of elution buffer (50 mM sodium phosphate, 0.3 M sodium chloride, 250 mM imidazole pH 8.0). The eluate was concentrated using the Amicon Ultra-15 (EMD Millipore) filters and protein concentration was determined by a Bradford assay, and the protein quality was analysed by Western blotting.

Supernatants from HEK293T cultures were concentrated 5-fold by tangential flow and protein was purified using His60 Ni Superflow (Clontech) in accordance with the manufacturer’s instructions. Purified protein was dialysed against PBS and quantified using a Bradford assay.

### Glycan analysis

PNGase F and O-Glycosidase was purchased from NEB. Briefly, 2 μg of purified S1 protein was added with 1 μL of 10X glycoprotein denaturing buffer (NEB) in total of 10 μL of reaction volume and denatured at 95 °C for 5 min. Then, the mixture was chilled on ice for 30 s followed by centrifugation for 10 s at 10,000 X g. Reaction volume was increased to 20 μL by adding 2 μL 10X GlycoBuffer (NEB), 2 μL 10 % NP40, water and 1 μL of enzyme PNGase F or 2 μL of O-glycosidase and incubated at 37 °C for 1 h. The extent of glycosylation was analyzed by mobility shift on SDS-PAGE followed by Western blotting.

### Western blotting

Protein samples were heated in the SDS sample loading buffer (0.375 M Tris pH 6.8, 12 % SDS, 60 % glycerol, 0.6 M DTT, 0.06 % bromophenol blue) at 95 °C for 5 min. Samples were separated by electrophoresis in 10 % SDS-PAGE followed by transfer of proteins onto nitrocellulose membrane in Towbin’s buffer (0.025 M Tris, 0.192 M glycine, 20 % methanol) at 4 °C for 1 h at 100 V. The membrane was blocked with Blocker Blotto (Thermo Scientific) at room temperature for 1 h. The membrane was incubated in anti-His rabbit antibody (1:2000) in Tris-buffered saline (0.1 M Tris, 0.9 % NaCl) added with 0.1 % Tween-20 and 1 % skim milk at 4 °C on orbital shaker overnight. Membrane was washed three times in TBST, and alkaline phosphate goat anti-rabbit IgG antibody (1:5000) was added and incubated at room temperature for 1 h on orbital shaker. Unbound antibody was washed by three TBST washes and bands were visualized using an AP Conjugate Substrate Kit (BioRad).

### Pig immunization

All the animal experiments were performed in the animal containment level 3 laboratories of VIDO-InterVac. All pigs were maintained and euthanized as per the protocol, approved by the University of Saskatchewan’s Animal Research Ethics Board and adhered to the Canadian Council on Animal Care guidelines for humane animal use. Two commercial crossbred pregnant sows were used in this study. The sows were vaccinated intramuscularly on both sides of neck with either purified S1 protein (400 μg per dose) or saline mixed with TriAdj adjuvant [28] three times, 14 days apart (days 0, 14 and 28). Blood samples were collected for serum on days 0, 11, 28 and 35. Farrowing was induced at 16 days after the last vaccination. Colostrum samples were collected on the day of farrowing. Piglets were allowed to suckle their dams, and on the 4th day of their life they were orally challenged with live PEDV (3×10^2^ TCID_50_ per piglet). Clinical signs, diarrhea, death and weight of challenged piglets were monitored daily throughout the study. Blood samples of all the piglets were collected on the day of challenge for analysis. Rectal swabs were collected from each of the piglets daily for analyzing the presence of viral RNA by qRT-PCR.

### Elisa

Immulon 2 HB 96-well plates (ThermoFisher Scientific) were coated overnight with 0.1 ml/well of 0.5 μg/ml purified recombinant S1 protein. The plates were washed five times in phosphate buffer saline (PBS) containing 0.05 % Tween 20. Sera serially diluted in assay diluent buffer (0.1 M PBST, 0.05 % Tween-20, 1 % fish gelatin) were added to respective wells. After a 2 h incubation, the plates were washed, and 1/3000 diluted HRP-conjugated anti-pig IgG antibodies were added. After 1 h incubation, the plates were washed, and developed with 1-Step Ultra TMB-ELISA substrate solution (ThermoFisher Scientific). The reaction was stopped by adding 30 μL of 2 M sulphuric acid to each well, and optical density values were measured at 450 nm using an ELISA plate reader.

A colostrum sample (1 mL) collected on the day of farrowing was treated with 30 μL rennet (5 mg/mL, Sigma-Aldrich). The colostrum was then incubated at 37 °C for 1 h. Once solidified, the whey was separated by centrifugation at 6000 x g for 20 min. Whey samples were diluted in PBS containing 0.05 % Tween 20 and 1 % casein and applied at four-fold dilution on ELISA plate coated with the purified S1 protein. Plate was incubated at room temperature for 2 h. The plates were washed six times with water between each step. Mouse anti-pig IgA (AbD Serotec) was applied to the plate at 1:300 dilution and incubated for 1 h. Donkey anti-mouse HRP conjugate (Jackson Immunoresearch) was applied at 1:5000 dilution and incubated for 1 h, and 1-Step Ultra TMB-ELISA substrate solution (ThermoFisher Scientific) was applied to the plates to develop the reaction. Then, the reaction was stopped and red as described above.

### Serum neutralization

The presence of PEDV-specific neutralizing antibodies in serum of sows and piglets was determined using a serum neutralizing (SN) test. Briefly, serum samples were diluted 2-fold and mixed with an equal volume of 200 TCID_50_ of PEDV in each well. After a 1 h incubation at 37 °C, 100 μL of virus-serum mix was added to 96-well microtiter plate with a confluent monolayer of Vero 76 cells. The cells were incubated for 3 h at 37 °C in 5 % CO_2_ and unbound virus particles were removed with two washes of DMEM. Then, 100 μL DMEM supplemented with trypsin (2 μg/mL) was added to the cells and incubated for 1 h at 37 °C in 5 % CO_2_. Thereafter, 100 μL DMEM containing trypsin (2 μg/mL) and albumin (0.2 %) were added to each well and the cells were incubated for the period of 7 days at 37 °C in 5 % CO_2_. The virus neutralizing antibody titers were expressed as the reciprocal of the highest serum dilution that showed no CPE in the cells. PEDV positive and negative control sera were also included in the tests.

### Viral RNA isolation and qRT-PCR

Piglet fecal swabs were collected in 0.5 ml DMEM on every day post-infection and stored at -80 °C. RNeasy Plus Kit (Qiagen) was used for RNA isolation from the faecal swab samples. qRT-PCR was conducted in two steps: cDNA synthesis and PCR reactions. cDNA synthesis was performed with 1 μL (50 ng/μL) random hexamers, 1 μL of 10 mM dNTPs, and RNA in 13 μL volume and heated at 65 °C for 5 min and chilled on ice followed by addition of 4 μL of 5X First-strand buffer, 1 μL of 0.1 M DTT and 1 μL of RNaseOUT (ThermoFisher Scientific) and 1 μL of SuperScript III enzyme (ThermoFisher Scientific) in final volume of 20 μL. The reaction conditions include 25 °C for 5 min, 50 °C for 60 min and 70 °C for 15 min. The PCR reaction was performed in a total volume of 20 μL with 2 μl cDNA using Power SYBR green Master Mix (Qiagen); primers (5’-GCAACAACAGGTCCAGATCTC-3’ and 5’-CTCCACGACCCTGGTTATTTC-3’) were present at 0.5 μM. PCR cycling conditions were: 95 °C for 10 min and 41 cycles of 95 °C for 15 s, 60 °C for 1 min.

### Statistical analysis

All data were analyzed using the GraphPad Prism (Version 6) software. Differences between two groups were assessed using unpaired two-tailed *t*-test. Differences were considered significant if *P* < 0.05. Survival curves were created using the product limit method of Kaplan and Meier, and comparison of the curves was done using the logrank test.
